# Is the femoral component flexion affected by the sagittal femoral shaft bowing in conventional intramedullary guided TKA?

**DOI:** 10.1186/s13018-021-02822-7

**Published:** 2021-12-04

**Authors:** Xiaofeng Zhang, Qianjin Wang, Xingquan Xu, Dongyang Chen, Zhengyuan Bao, Yao Yao, Dengxian Wu, Bin Wang, Zhihong Xu, Qing Jiang

**Affiliations:** 1 Division of Sports Medicine and Adult Reconstructive Surgery, Department of Orthopedic Surgery, Nanjing Drum Tower Hospital Clinical College of Nanjing Medical University, 321 Zhongshan Road, Nanjing, 210008 Jiangsu People’s Republic of China; 2 Division of Sports Medicine and Adult Reconstructive Surgery, Department of Orthopedic Surgery, Nanjing Drum Tower Hospital, The Affiliated Hospital of Nanjing University Medical School, 321 Zhongshan Road, Nanjing, 210008 Jiangsu People’s Republic of China

**Keywords:** Sagittal, Coronal, Femoral bowing angle, Femoral shaft bowing, TKA

## Abstract

**Background:**

The aim of the present study was to investigate the influence of sagittal femoral bowing on sagittal femoral component alignment, and whether there was correlation between sagittal femoral component alignment and coronal femoral component alignment.

**Methods:**

We retrospectively reviewed 77 knees in 71 patients who had undergone primary TKA for advanced osteoarthritis. All surgeries were performed by using a standard medial parapatellar approach. The osteotomy was performed with a conventional technique using an intramedullary rod for the femur and a mechanical extramedullary guiding system for the tibia. All patients enrolled in the study were evaluated with full-length lower extremity load-bearing standing scanograms, and the patients had preoperative and postoperative radiographs of the knees. Coronal femoral bowing angle (cFBA), sagittal femoral bowing angle (sFBA), and postoperatively, mechanical tibiofemoral angle of the knee (mTFA), β angle (femoral component flexion angle) were measured. The radiographic results of both groups were compared using Student's *t* test. A two-sided Pearson correlation coefficient was obtained to identify the correlations between FBA in the coronal and sagittal planes, as well as FBA and age or BMI, sFBA and β angle, cFBA and mTFA. Comparison of FSB incidence between different genders was made using Chi-square test. The *p* value < 0.05 indicates a statistically significant difference.

**Results:**

The mean sFBA, cFBA, β angle, mTFA were 9.34° ± 3.56°(range 1°–16°), 3.25° ± 3.79°(range − 7° to −17°), 3.91° ± 3.15°(range − 1° to −13°), 0.60° ± 1.95°(range − 3° to −6°), respectively. There was no correlation between age and sFBA (CC = 0.192, *p* = 0.194) or cFBA (CC = 0.192, *p* = 0.194); similarly, there was no correlation between age and sFBA (CC = 0.067, *p* = 0.565) or cFBA (CC = 0.069, *p* = 0.549). The sFBA was correlated with cFBA and β angle (CC = 0.540, *p* < 0.01; CC = 0.543, *p* < 0.01, respectively), and the cFBA was correlated with mTFA (CC = 0.430, *p* < 0.01). There was no significant difference (*p* = 0.247) of cFBA between the patients with sFSB and the patients without sFSB.

**Conclusions:**

The current study showed that the sFBA was correlated with cFBA in the patients undergoing TKA and the patients with sFSB usually presented non-cFSB. We also found that sFSB could affect the femoral component alignment in the sagittal plane and cFSB could affect the femoral component alignment in the coronal plane. The sFBA or cFBA was not correlated with age, BMI, or gender.

## Background

Total knee arthroplasty (TKA) relieves pain, restores function, is one of the most successful treatment for advanced knee osteoarthritis [1, 2]. To maximize the success and longevity of a TKA, the surgeon must implant the components in the optimal position to re-create the knee anatomic alignment [3]. The TKA often sacrifices the natural anatomy of the knee, and nearly 15% of patients are not satisfied after TKA [2]. There are some factors affecting the outcome of TKA, and one of which is alignment of components [4]. Malalignment of components can cause premature component loosening, abnormal wear [5], and patellofemoral complications such as anterior knee pain [6]. Sagittal alignment of the femoral component may influence the clinical results of TKA in various ways [7]. If a femoral component is placed in an overly flexed position, the extension or polyethylene postwear resulting from impingement between the anterior part of the polyethylene insert and the intercondylar box in TKA [8]. When a femoral component is implanted in hyperextension relative to the femur, the surgeon may create a notch in the anterior femoral cortex, which may increase the prospective risk of a supracondylar fracture [9]. Sagittal alignment of the femoral component is determined by several factors such as prosthesis design, sagittal femoral bowing angle (sFBA), the entry point of intramedullary guide, and reamer diameter [4]. Some researches have shown that sagittal femoral bowing can affect the position of femoral component in sagittal plane [10, 11]. Due to the sagittal curvature of the femur, the extended distal femoral resection with conventional intramedullary technique may lead to error in the sagittal plane, especially in the patients with large sFBA. A cutting mistake of the distal femur may cause selecting larger or smaller prosthesis, and these errors are attributable to the difficulty in accurately cutting the bone. Errors of sagittal alignment of the distal femur may affect the sizing of the prepared bone. The femoral component should be located anteriorly when the distal femur is cut in a extended position to avoid anterior notching and be located posteriorly to avoid the impingement when the distal femur is cut in a flexed position [1]. Unlike numerous studies of coronal femoral component alignment [12–15], few studies have explored the clinical implications of femoral component rotation in the sagittal plane. The studies have suggested femoral component sagittal alignment could be affected by the presence of femoral bowing [8, 10].

The aim of the present study was to investigate the influence of sagittal femoral bowing on sagittal femoral component alignment, and whether there was correlation between sagittal femoral component alignment and coronal femoral component alignment.

## Materials and methods

### Demographics

We retrospectively reviewed 77 knees in 71 patients who had undergone primary TKA for advanced osteoarthritis with using the same treatment protocol from September 2017 to February 2019 in Nanjing Drum Tower Hospital, The Affiliated Hospital of Nanjing University Medical School. Demographic data (Table 1) collected included patient BMI, age, gender. There were 58 female and 13 male patients. Mean age in the patients was 69.4 years (range 54–85 years), and body mass index was 27.7 kg/m^2^ (range 17.6–40.8 kg/m^2^). Patients meeting the following criteria were excluded from analysis: (a) a history of femoral or tibial fracture or osteotomy around the knee; (b) the presence of a congenital anomaly in the femur or tibia; (c) a history of prior knee or hip arthroplasty; (d) a diagnosis other than primary osteoarthritis; (e) the absence of radiographs pre-operation or postoperation. No patients were recalled specifically for this study; all data were obtained from medical records and radiographs.

**Table 1:** Patient demographics

	Total
Gender	
Male	13
Female	58
Age (years)	69.4 ± 7.5
BMI (kg/m^2^)	27.7 ± 4.4

### Surgical procedure

All surgeries were performed by three surgeons (Dr. Xu ZH, Dr. Chen DY, and Dr. Shi DQ) experienced in knee arthroplasty using a standard medial parapatellar approach. The patients were implanted with fixed-bearing posterior-stabilized prosthesis (Genesis II; Smith&Nephew, Memphis, TN, USA). The procedure was performed through a anterior longitudinal incision of 15–20 cm in length with a medial parapatellar approach. The osteotomy was performed with a conventional technique using an intramedullary rod for the femur and a mechanical extramedullary guiding system for the tibia. The planned position of the tibial component was at a valgus angle of 90° in the coronal plane and a flexion angle of 87° in the sagittal plane. To achieve a distal femoral bone cut perpendicular to the mechanical axis of the femur, the angle of the distal femoral cutting block was determined according to the measured angle on a long-leg weight-bearing split scanogram between the femoral mechanical axis and the femoral anatomical axis. The patella was resurfaced and all prostheses were fixed with cement. Ambulation with a walking aid was initiated on the second postoperative day. Typically, patients were discharged to home or to a rehabilitation center 3 days after surgery.

### Radiological evaluation

All patients enrolled in the study were evaluated with full-length lower extremity load-bearing standing scanograms. The patients had preoperative and postoperative radiographs of the knees including anteroposterior (AP) and lateral, as described in detail in a previous publication [16]. The radiographs obtained by using a GE Computed Radiography System (GE Health Co. Ltd., 646HD, USA) and Picture Archiving Communication System (PACS, FIRSTECH, Hefei, Anhui, China). This direct lateral full-length lower extremity radiograph technique was used to reduce rotational effect which was described by Minoda et al. [17]. The radiographs were performed with the patient standing on the involved leg in neutral rotation with the knee fully extended and the uninvolved leg flexed at the hip and knee.

**Fig. 1:**
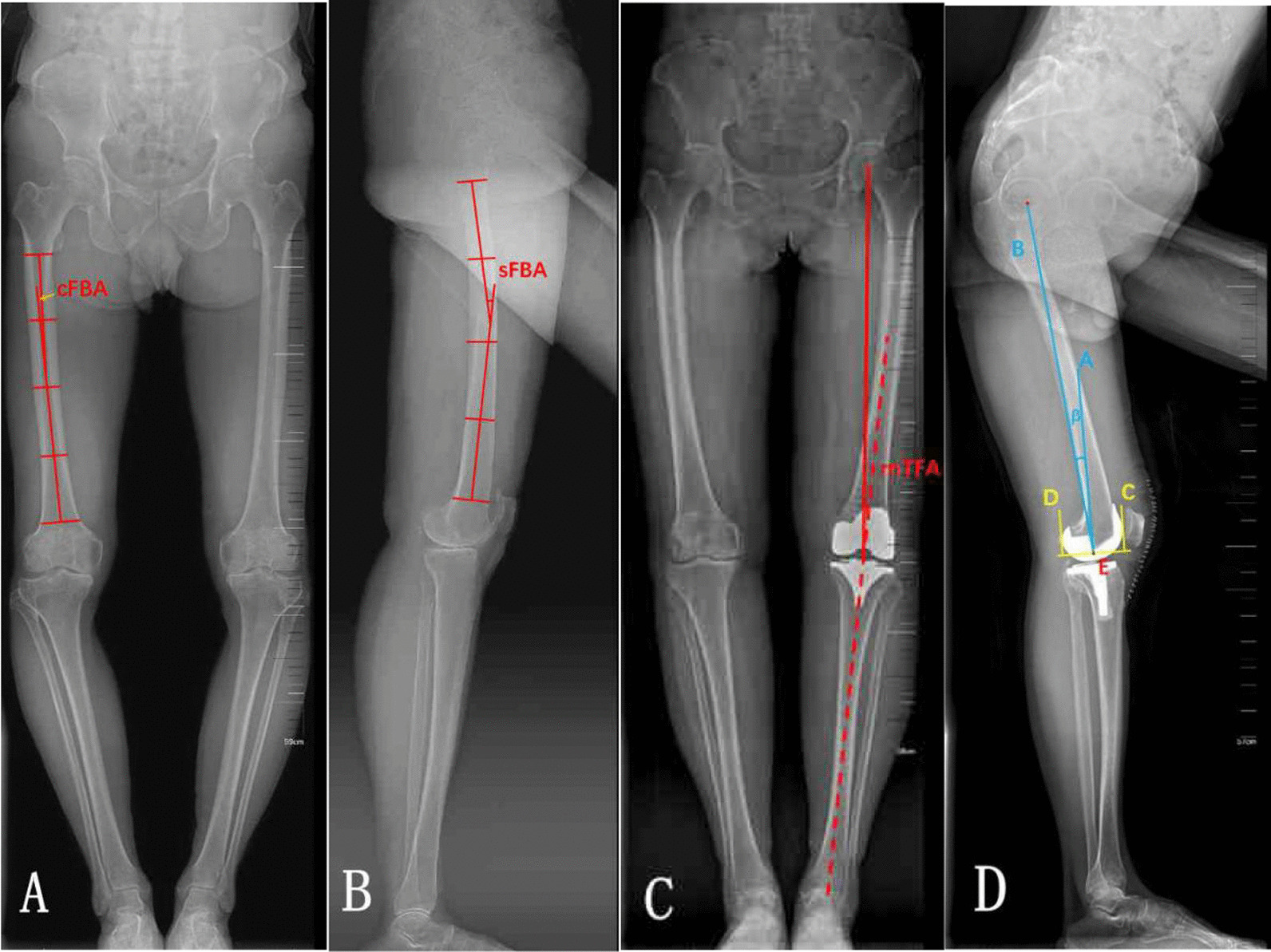
**A** The femoral shaft was divided into four equal parts in the coronal plane. The proximal end of the diaphysis was the lower border of the lesser trochanter, and the distal end was the junction between the shaft and the condylar region. The angle between the midlines drawn in the proximal and distal quarter segments was defined as cFBA. **B** The femoral shaft was divided into four equal parts in the sagittal plane, and the angle between the midlines drawn in the proximal and distal quarter segments was defined as sFBA. **C** Mechanical tibiofemoral angle of the knee (mTFA) was defined as the angle formed by the intersection between the mechanical axes of the femur and the tibia. **D** β angle (femoral component flexion angle) was defined as the angle between the A line and B line. C line was defined as the anterior condyle tangent line perpendicular to the distal femur resection in the sagittal plane, D line was defined as the posterior condyle tangent line perpendicular to the distal femur resection in the sagittal plane, and the point E was defined as the center in distal femur resection between C line and D line. A line was defined as the perpendicular line to distal femur resection from the point E, and B line was from femur head center to point E

The operated limbs were evaluated. Preoperatively, in the AP radiographs, the femoral shaft was divided into four equal parts in the coronal plane. The proximal end of the diaphysis was the lower border of the lesser trochanter, and the distal end was the junction between the shaft and the condylar region. The angle between the midlines drawn in the proximal and distal quarter segments was defined as coronal femoral bowing angle (cFBA) (Fig. 1A) [18–21]. Lateral angulation of > 5° in the coronal plane was defined as lateral FSB, also called coronal femoral shaft bowing (cFSB). As similar as sagittal plane, in the lateral radiographs, the femoral shaft was divided into four equal parts in the sagittal plane, and the angle between the midlines drawn in the proximal and distal quarter segments was defined as sagittal femoral bowing angle (sFBA) (Fig. 1B). Anterior angulation of > 11° in the sagittal plane was defined as anterior femoral shaft bowing (FSB), also called sagittal femoral shaft bowing (sFSB).

Postoperatively, a standing whole-limb AP and lateral radiograph taken was used to measure mechanical tibiofemoral angle [22] for coronal femoral prosthesis alignment and sagittal femoral angle [7] for sagittal femoral prosthesis alignment. As a surrogate of overall limb alignment, mechanical tibiofemoral angle of the knee (mTFA) was defined as the angle formed by the intersection between the mechanical axes of the femur (the line from the femoral head center to the femoral intercondylar notch center) and the tibia (the line from the ankle talus center to the center of tibial spine tips) (Fig. 1C) [22]. A negative value was given to knees in varus alignment. β angle (also was called femoral component flexion angle) was defined as the angle between the A line and B line (Fig. 1D) [23]. C line was defined as the anterior condyle tangent line perpendicular to the distal femur resection in the sagittal plane, D line was defined as the posterior condyle tangent line perpendicular to the distal femur resection in the sagittal plane, and the point E was defined as the center in distal femur resection between C line and D line. A line was defined as the perpendicular line to distal femur resection from the point E, and B line was from femur head center to point E. A negative value was given to femoral component in hyperextension.

A radiological evaluation was performed by two independent observers (Wang QJ and Bao ZY). The reliabilities of assessments of all radiographic measurements were evaluated using intraclass correlation coefficients (ICCs). The ICCs of the observers reliabilities of all measurements were 0.910 and 0.938.

### Statistical analysis

The patients were divided into two groups: those with or without anterior femoral bowing (sFSB), then the patients were also divided into two groups: those with or without lateral femoral bowing (cFSB). Data were analyzed using SPSS 19.0 (SPSS Inc., Chicago, IL, USA). All the data were expressed as the mean ± standard deviation (SD) and range. The radiographic results of both groups were compared using Student's *t* test. A two-sided Pearson correlation coefficient was obtained to identify the correlations between FBA in the coronal and sagittal planes, as well as FBA and age or BMI, sFBA and β angle, cFBA and mTFA. Comparison of FSB incidence between different genders was made using Chi-square test. The *p* value < 0.05 indicates a statistically significant difference.

## Results

The incidence of sFSB was 31.17% (24 knees) and the incidence of cFSB was 27.27% (21 knees). There were 5 patients (7.04%) with both sFSB and cFSB. There was no correlation (Table 2) between gender and sFSB (^2^ = 0.323, *p* = 0.570), and there was no correlation between gender and cFSB (^2^ = 1.051, *p* = 0.305). The mean sFBA, cFBA, β angle, mTFA were 9.34° ± 3.56°(range 1°–16°), 3.25° ± 3.79° (range − 7° to −17°), 3.91° ± 3.15° (range − 1° to −13°), 0.60° ± 1.95° (range − 3° to −6°), respectively. There was no correlation (Table 3) between age and sFBA (CC = 0.192, *p* = 0.094) or cFBA (CC = 0.062, *p* = 0.595); similarly, there was no correlation between BMI and sFBA (CC = 0.067, *p* = 0.565) or cFBA (CC = 0.069, *p* = 0.549). The sFBA was correlated with cFBA and β angle (CC = 0.540, *p* < 0.01; CC = 0.543, *p* < 0.01, respectively), and the cFBA was correlated with mTFA (CC = 0.430, *p* < 0.01). The correlation between sFBA and cFBA was poor (CC = 0.120, *p* = 0.578) in the patients with sFSB (Table 4), and there was no significant difference (*p* = 0.247) of cFBA between the patients with sFSB and the patients without sFSB (Table 5). There was significant difference (*p* < 0.01) of β angle between the patients with sFSB and the patients without sFSB, and there was significant difference (*p* = 0.014) of mTFA between the patients with cFSB and the patients without cFSB (Table 6).

**Table 2:** The correlation between gender and sFSB or cFSB

	sFSB	non-sFSB	*χ*^2^	*p* value	cFSB	non-cFSB	*χ*^2^	*p* value
Male	3	10			2	11		
Female	18	40	0.323	0.570	17	41	1.051	0.305

There was no correlation between gender and sFSB or cFSB

**Table 3:** Pearson correlation coefficients between between age, BMI, sFBA, cFBA, β angle and mTFA

		Age	BMI	sFBA	cFBA	β angle	mTFA
Age	Pearson correlation coefficient	1	−.017	.192	.062	.278*	.052
	Significance (two-sided)		.880	.094	.595	.014	.656
BMI	Pearson correlation coefficient	−.017	1	.067	.069	−.069	−.050
	Significance (two-sided)	.880		.565	.549	.553	.663
sFBA	Pearson correlation coefficient	.192	.067	1	.540**	.543**	.193
	Significance (two-sided)	.094	.565		.000	.000	.093
cFBA	Pearson correlation coefficient	.062	.069	.540**	1	.241*	.430**
	Significance (two-sided)	.595	.549	.000		.035	.000
β angle	Pearson correlation coefficient	.278*	−.069	.543**	.241*	1	.062
	Significance (two-sided)	.014	.553	.000	.035		.589
mTFA	Pearson correlation coefficient	.052	−.050	.193	.430**	.062	1
	Significance (two-sided)	.656	.663	.093	.000	.589	

Pearson correlation coefficients between age, BMI, sFBA, cFBA, β angle and mTFA (n = 77)

*p < 0.05; **p < 0.01

**Table 4:** The correlation between sFBA and cFBA in the patients with sFSB

	Mean		sFBA	cFBA
sFBA	13.54° ± 1.32°	Pearson correlation coefficient	1	.120
		Significance (two-sided)		.578
cFBA	2.50° ± 4.27°	Pearson correlation coefficient	.120	1
		Significance (two-sided)	.578	

Pearson correlation coefficients between sFBA and cFBA in the patients with sFSB (n = 24)

**Table 5:** Comparison of cFBA between the patients with sFSB and the patients without sFSB

	*n*	Mean	Variance equality test		*T* test	
			*F*	Sig	Sig. (two-sided)	95% CI
cFBA (sFSB)	24	2.50° ± 4.27°	1.024	.315	.247	−2.94 to −.769
cFBA (non-sFSB)	53	3.58° ± 3.54°				

There was no significant difference of cFBA between the patients with sFSB and non-sFSB

**Table 6:** Comparison of β angle between the patients with sFSB and the patients without sFSB, comparison of mTFA between the patients with cFSB and the patients without cFSB

	*n*	Mean	*p* value		*n*	Mean	*p* value
β angle (sFSB)	24	6.00° ± 3.05°	.000	mTFA (cFSB)	21	1.48° ± 2.16°	0.014
β angle (non-sFSB)	53	2.96° ± 2.74°		mTFA (non-cFSB)	56	0.28° ± 1.77°	

There was significant difference of β angle between the patients with sFSB and non-sFSB, and there was significant difference (*p* = 0.014) of mTFA between the patients with cFSB and the patients without cFSB

## Discussion

The most important finding of the present study was that femoral anterior bowing was an influential factor for implant positioning in TKA with conventional femur osteotomy. Many authors have concluded that correct alignment of the prosthesis was correlated with clinical success in TKA [11]. Moreover, improper positioning of components during surgery may lead to early loosening of the implant due to impingement between the cam and the post in posterior stabilized systems [24]. Extensive study has been performed on ideal coronal alignment of the femoral component in total knee prostheses [3]. In contrast, there are limited studies addressed sagittal alignment of femoral component. In general, there is no consensus on sagittal mechanical axis of the alignment of femur and femoral component [4].

Lasam et al. [22] found that the proportion of knees with cFSB of was 42.2% in the TKA group in women, and Yasushi Akamatsu et al. [18] reported that the proportion of knees with lateral FSB of angulation of > 5° was 37.8%, which was similar to our finding in this study. It is controversial whether age, BMI, gender were the factors associated with sFSB or cFSB. In this study, we found that FSB was not associated with age, BMI, or gender. Although some researchers thought sFSB was associated with BMI, and anterior FSB and age were correlated in women [18, 25], there was no clear conclusion. Yehyawi et al. [8] demonstrated that large variances of sagittal femoral bowing and the taller and heavier patients had less distal bowing, and men had greater proximal and less distal bowing than women. Egol et al. [26] stated that there was no correlation between age and anterior FSB. Walensky [27] shows that American-Indians exhibited greater anterior curvature than Caucasians and African-Americans, and the femora of Eskimos was more closely related to American-Indians. Tang et al. [28] studied the alignment of femur in sagittal plane in Chinese population; they demonstrated that the distal one-third of the femur was not just curved anteroposteriorly, but it was more bowed than the middle and upper segments. The finding of Kim et al. [12] study was that femoral anterior bowing was an influential factor for implant positioning in conventional TKAs. If the femoral component is too flexed, the impingement of the femoral cam on the anterior aspect of the polyethylene post can cause accelerated wear of the post. Therefore, it should draw more surgeons’ attention to sFSB, especially in the East-Asians. Tang et al. [28] recommend that the patients with obvious femoral bowing at the distal femur as seen on the preoperative long film should be used with caution when performing intramedullary guide in TKA. Similar results were found by Bao et al. [19].

In this study, we found that there was a significant correlation between sFBA and cFBA, and little literature discussed the correlation between sFBA and cFBA. We conjectured the relationship between sFBA and cFBA may be regulated by femur growth and development. However, the patients with sFSB usually presented non-cFSB. According to our data, we observed that cFBA was not correlated with sFBA in the patients with sFSB, and cFBA in the patients with sFBA showed no significant difference when comparing with non-sFBA. Our data suggested that there was no correlation between coronal femoral shaft bowing and sagittal femoral shaft bowing in the OA patients with undergoing TKA.

**Fig. 2:**
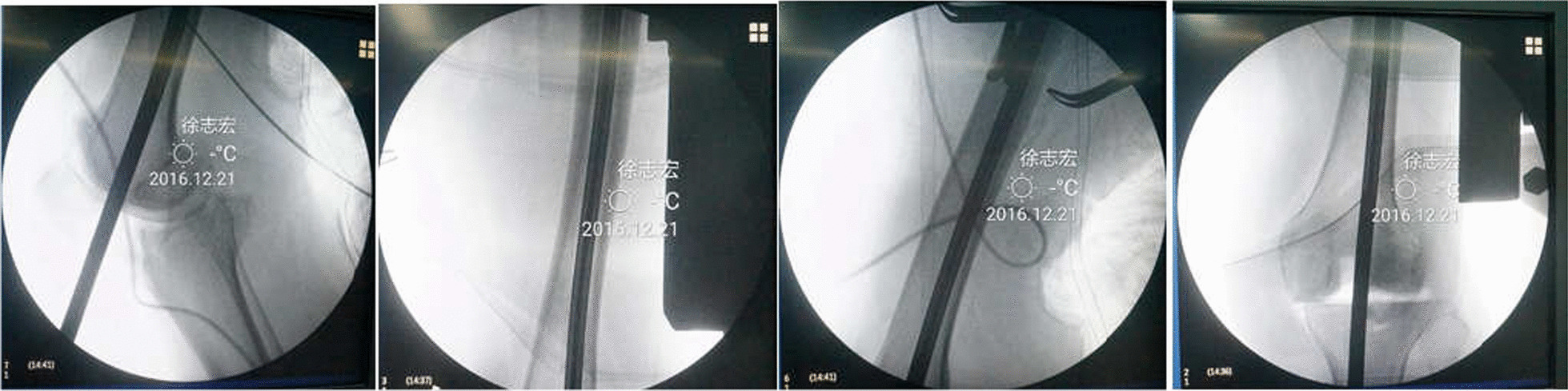
The intramedullary guide rod was pushing into a femur

The implant alignment is an issue of high importance in TKA. Young-Hoo Kim et al. [29] studied 3018 patients who underwent total knee arthroplasty, and they thought that when total knee components in the position of: femoral coronal alignment, 2°–8° valgus; femoral sagittal alignment, 0°–3°; tibial coronal alignment, 90°; tibial sagittal alignment, 0°–7°; femoral external rotational alignment, 2°–5°; tibial external rotational alignment, 2°–5°; and overall anatomical knee alignment at an angle of 3°–7.5° valgus, the survival rate of the prosthesis could improve. Whether coronal femoral shaft bowing or sagittal femoral shaft bowing, the femoral component alignments in the coronal plane or sagittal plane were affected [7, 22]. The view was further confirmed in our study. Comparison between β angle in the sFSB patients and the non-sFSB patients was significant difference; also, there was significant difference between mTFA of the patients with cFSB and the patients with non-cFSB. As mentioned in many previous publications [20, 22, 30–32], the presence of coronal femoral shaft bowing may lead to varus orientation of the femoral component during the implantation with using intramedullary guiding system. Similarly, we found that the cFBA was correlated with mTFA. Coronal variations in femoral shape may result in a distal cut which was not perpendicular to the femoral mechanical axis. Accurate distal femoral resection is challenging because it was difficult to determine the mechanical axis during surgery. The standard practice to determine distal cutting angle referenced off the femoral intramedullary guide was usually to measure the angle between the anatomical and mechanical femoral axis on preoperative radiographs [12]. The position of TKA implant significantly affects the outcome of joint replacement in the coronal plane [33–35]. Although femoral component alignment has been thoroughly studied, the importance of femoral sagittal bowing in TKA has not been widely studied [11]. No clear safety margin has been documented in the sagittal plane. Sagittal femoral shaft bowing should be considered in TKA because the axis of the distal femur was more flexed than the sagittal femoral mechanical axis when sagittal femoral bowing angle increased [36]. Kazemi et al. [4] studied 25 patients who underwent TKA using cruciate retaining knee prosthesis, they found that there was a significant positive correlation between femoral component flexion in relation to mechanical axis and amount of sagittal femoral bowing, amount of flexion in relation to mechanical axis and DACL was 8.4° ± 2.9° (range: 4°–14.3°) and 1.7° ± 0.9° (range: 0°–3°). In our study, we found that the sFBA was correlated with β angle (CC = 0.543, *p* < 0.01) and cFBA was correlated with β angle (CC = 0.241, *p* = 0.035). On the other hand, femoral component alignment in sagittal plane may be affected by the coronal femoral bowing angle. As known, alignment of the femoral component in sagittal plane is determined by some factors including the entry point of intramedullary guide, depth of guide insertion, sagittal femoral shaft bowing, implant design [4]. Furthermore, sagittal femoral position had a significant influence on patellar kinematics [5] and increased femoral component flexion decreased the flexion gap and altered condylar lift-off and tibiofemoral kinematics [37, 38]. Sebastien Lustig et al. [39] demonstrated the angle between distal femoral cut in the sagittal plane and the mechanical axis more than 3.5° was an independent risk factor for clinically detectable flexion contracture. Consequently, Hiroyuki N et al. [1] advocated that upsizing or downsizing of femoral component could occur if the femoral osteotomy was performed in at least 3° extension or flexion. Usually, distal femoral cutting error in the sagittal plane which lead to hyperflexion of femur component position was due to using intramedullary alignment guide in the femur with sagittal femoral shaft bowing, as shown in Fig. 2. Computer navigation may achieve more accurate alignment [21, 40], and the previous studies showed that the use of navigation improves alignment. The overall incidence of mechanical tibiofemoral angle outliers was lower in the navigation group (15.4% vs 24.9%) [22]. However, there was controversial, Chen et al. [7] found that navigated TKAs resulted in a higher risk of hyperextension of the femoral components, and Jae Han Ko et al. [36] found that the femoral implant position was more extended in navigated TKAs than in conventional TKAs. Xu et al. [23] invented an extramedullary device which was easy and convenient, and this instrument could help the surgeons perform TKAs with achieving better alignment in both coronal and sagittal planes.

Several limitations in this study must be acknowledged. First, the main problem was insufficient sample size during the study period about how to determine and measure sagittal alignment of the components to provide the statistical power. Second, we used only one conventional instrumentation system (Genesis II), and different knee prosthesis may produce different results. Third, this was a radiological study and we did not assess any relation between alignment and function.

In summary, we first assessed the correlation between sFBA and cFBA; in the study, we found that the sFBA was correlated with cFBA. Using conventional intramedullary alignment guide, sFSB will affect the femoral component alignment in the sagittal plane and cFSB will affect the femoral component alignment in the coronal plane. It was unclear whether there was any correlation between age, BMI, or gender and sFSB or cFSB. The patients with sFSB usually presented non-cFSB.

## Conclusions

The current study showed that the sFBA was correlated with cFBA in the patients undergoing TKA and the patients with sFSB usually presented non-cFSB. We also found that sFSB could affect the femoral component alignment in the sagittal plane and cFSB could affect the femoral component alignment in the coronal plane. The sFBA or cFBA was not correlated with age, BMI, or gender.

## Data Availability

The datasets used and/or analyzed during the current study are available from the corresponding author on reasonable request.

## Abbreviations

BMI: Body mass index
cFBA: Coronal femoral bowing angle
cFSB: Coronal femoral shaft bowing
cMA: Coronal mechanical axis
mTFA: Mechanical tibiofemoral angle of the knee
OA: Osteoarthritis
sFBA: Sagittal femoral bowing angle
sFSB: Sagittal femoral shaft bowing
TKA: Total knee arthroplasty
β angle: Femoral component flexion angle
